# Mechanisms of Litchi Response to Postharvest Energy Deficiency via Energy and Sugar Metabolisms

**DOI:** 10.3390/foods13142288

**Published:** 2024-07-20

**Authors:** Kunkun Zhao, Zhaoyin Gao, Mir Muhammad Nizamani, Meijiao Hu, Min Li, Xiaohui Li, Jiabao Wang

**Affiliations:** 1 Tropical Crops Genetic Resources Institute, Chinese Academy of Tropical Agricultural Sciences, Haikou 571101, China; kkzhao@catas.cn (K.Z.); gaozhaoyin@catas.cn (Z.G.); 2 Department of Plant Pathology, Agricultural College, Guizhou University, Guiyang 550025, China; mirmohammadnizamani@outlook.com; 3 Environment and Plant Protection Institute, Chinese Academy of Tropical Agricultural Sciences, Haikou 571101, China; humeijiao320@catas.cn (M.H.); liminhzs@catas.cn (M.L.); 4 Hainan Inspection and Detection Center for Modern Agriculture, Haikou 570100, China

**Keywords:** litchi, quality, 2,4-dinitrophenol, energy metabolism, sugar metabolism

## Abstract

In the post-harvest phase, fruit is inexorably subjected to extrinsic stressors that expedite energy expenditure and truncate the storage lifespan. The present study endeavors to elucidate the response strategies of litchi to the alterations of energy state caused by 2,4-Dinitrophenol (DNP) treatment through energy metabolism and sugar metabolism. It was observed that the DNP treatment reduced the energy state of the fruit, exacerbated membrane damage and triggered rapid browning in the pericarp after 24 h of storage. Furthermore, the expression of genes germane to energy metabolism (*LcAtpB*, *LcAOX1*, *LcUCP1*, *LcAAC1*, and, *LcSnRK2*) reached their peak within the initial 24 h of storage, accompanied by an elevation in the respiratory rate, which effectively suppressed the rise in browning index of litchi pericarp. The study also posits that, to cope with the decrease of energy levels and membrane damage, litchi may augment the concentrations of fructose, glucose, inositol, galactose, and sorbose, thus safeguarding the canonical metabolic functions of the fruit. Collectively, these findings suggest that litchi can modulate energy and sugar metabolism to cope with fruit senescence under conditions of energy deficiency. This study significantly advances the understanding of the physiological responses exhibited by litchi fruit to post-harvest external stressors.

## 1. Introduction

Litchi, a fruit indigenous to tropical and subtropical regions, is appreciated by consumers for its vibrant hues and distinctive palatability [1]. Nevertheless, harvesting severs the supply of water and nutrients from the tree to the litchi fruit, resulting in the energy consumption of pericarp being met only by the fruit’s own reserves. Under the influence of abiotic and biotic stresses, the postharvest litchi pericarp tends to brown rapidly [2]. This phenomenon commonly occurs during the transportation and sale of litchi, shortening its shelf life and acting as one of the main bottlenecks in the development of the litchi trade [3]. Extensive studies have shown that the main cause of pericarp browning is attributed to the overproduction of reactive oxygen species (ROS) and damage to the cellular membrane structure, both of which are associated with energy deficiency [4]. Therefore, the energy dynamics have been widely recognized as a key factor in regulating fruit browning [5]. It is widely confirmed that abiotic/biotic stressors stimulate energy expenditure, intensifying respiratory activity and ROS generation [6,7], which upon surpassing the plant’s tolerance threshold, result in irreparable cellular membrane damage, expediting cellular senescence or demise [8]. For example, the decrease in energy status under external stress exacerbates the deterioration of fruit quality, such as bananas, longans, peaches, and pears [9,10,11,12]. Accordingly, the browning process of the pericarp can be significantly inhibited by applying appropriate treatments to enhance the energy state of litchi [4,13,14]. Given that current postharvest treatments have not fully resolved the issue of litchi pericarp browning [3], it is necessary to conduct in-depth research on the mechanisms by which litchi respond to pericarp browning under energy-deficient conditions. This will aid in understanding the fruit’s physiological responses and is crucial for developing more effective postharvest preservation techniques.

The types and concentrations of sugars determine the quality and flavor profiles of fruit, directly affecting the consumers’ sensory experience [15,16]. Additionally, multiple studies have confirmed that sugars, acting as energy substrates and signaling molecules, are vital in controlling fruit ripening, senescence, and stress responses [17,18]. Currently, some measures to increase sugar content or utilization efficiency in cells are widely applied in postharvest fruit treatments. For instance, methyl jasmonate treatment of Pitaya fruit promoted the utilization and transformation of sugar, leading to higher energy states, and enhanced the accumulation of phenolics in Pitaya fruit in response to wounding stress [19]. Exogenous glycine betaine regulates soluble sugar metabolism, thereby affecting energy levels and reducing pericarp browning in pear fruit [20]. In addition, extensive studies have confirmed that sugar metabolism plays a crucial role in the stress response of postharvest horticultural products, such as peach, pear, and apricot [21]. In the postharvest studies of litchi, variations in sugar content were typically employed as a metric for evaluating the quality during storage [22,23,24]. However, the mechanisms through which litchi responds to energy deficiency via sugar metabolism after harvest remain unclear.

2,4-Dinitrophenol (DNP), as a highly effective mitochondrial uncoupler, can cause protons (H^+^) to bypass ATP synthase and return directly to the matrix through the mitochondrial membrane, thereby reducing the efficiency of ATP synthesis [25]. This characteristic makes DNP an ideal reagent for studying plant responses under energy-deficient conditions. For example, Kılıç and Dumlupınar studied the cold resistance mechanisms of plants under low-energy conditions by treating corn seeds with DNP [26]. Shu et al. used DNP to treat apples to study the fruit’s stress responses to vibrations during simulated transportation [7]. This study explored the effects of DNP treatment on postharvest browning, energy and sugar metabolism in litchi, aiming to provide a deeper understanding of the regulatory mechanisms that underpin postharvest fruit responses to energy deficiency.

## 2. Materials and Methods

### 2.1. Plant Material and Sample Handling

Litchi fruit (*Litchi chinensis* Sonn. cv. ‘A4 Wuhe’) was sourced from an orchard situated in Haikou, China. After harvest, all the fruits were refrigerated with ice packs and swiftly transported back to the laboratory. For the study, litchi fruit exhibiting 80% maturity, uniformity in size, and devoid of mechanical injuries or signs of pest infestation and disease were meticulously chosen as experimental specimens. After disinfection with a prochloraz solution (0.1%, *v*/*v*), the litchi were randomly divided into two groups, each consisting of 600 fruits. The fruits in the experimental group were immersed in a 1.0 mM DNP solution, whereas the control group was submerged in distilled water, with both soaks enduring for a duration of 15 min [27]. After immersion, the pericarp of the fruits was dried using filter paper, and both groups of fruits were placed in fruit trays (20 fruits/tray). They were then stored under controlled conditions of 25 ± 1 °C temperature and 85 ± 5% relative humidity. The samples were tested at 0 h, 12 h, 24 h, 48 h, 72 h, and 96 h respectively. Pericarp browning and respiratory rate: 60 fruits were randomly selected each time, with 20 fruits per replicate. Membrane permeability analysis: 30 fruits were randomly selected each time, with 10 fruits per replicate. In addition, pericarp tissues were carefully separated from 10 fruits at each sampling and stored in liquid nitrogen tanks for subsequent analysis. Each parameter was measured three times to ensure the accuracy of the results.

### 2.2. Determination of Fruit Browning, Respiratory Rate and Membrane Integrity

The degree of pericarp browning in litchi was categorized into five distinct levels, as follows: 0 (no browning), 1 (≤25% browning area), 2 (25–50% browning area), 3 (50–75% browning area), and 4 (≥75% browning area). The calculation of browning index (BI) followed the method of Zhang and Quantick (1997) [28].

The respiratory rate of the fruit in both groups was quantified employing an O_2_/CO_2_ analyzer (PBI Dansensor CheckPoint 3, Mocon, Denmark), following the methodology described by Bai et al. (2022) [29]. The respiratory rate was expressed in units of µmol kg^−1^ s^−1^.

The membrane integrity was assessed by evaluating membrane permeability and malondialdehyde (MDA) content [29]. The membrane permeability of the litchi pericarp was ascertained using the procedure described by Liu et al. (2011) [30]. The results were expressed in percentages. MDA content was quantified following the procedure established in previous research [31], with the unit of measurement being µmol kg^−1^ fresh weight.

### 2.3. Determination of Energy State in Pericarp

The concentrations of adenosine triphosphate (ATP), adenosine diphosphate (ADP), and adenosine monophosphate (AMP) within the litchi pericarp were ascertained utilizing the methodology described by Zhang et al. (2017) [5]. These adenine nucleotides were quantified and expressed in terms of millimoles per kilogram of fresh weight (mmol kg^−1^ FW). The energy charge (EC) was computed employing the formula: ([ATP] + 0.5 × [ADP])/([ATP] + [ADP] + [AMP]).

### 2.4. Quantitative Analysis of Energy Metabolism-Associated Gene Expression

The procedures of total RNA extraction and cDNA synthesis were completed using 1.5 g litchi pericarp according to the protocol described by Tang et al. (2020) [32]. Total RNA was extracted and purified from each sample using the RNAprep Pure Plant Kit (DP432; TIANGEN Biotech, Beijing, China). The purified RNA was reverse transcribed to synthesize the first strand cDNA using the PrimeScript^®^ RT Master Mix Perfect Real Time (DRR036A; TAKARA, Dalian, China) according to the kit’s instructions. The primer sequences for energy metabolism-associated genes (ATP synthase β-subunit, *LcAtpB*; alternative oxidase 1, *LcAOX1*; mitochondrial uncoupling protein 1, *LcUCP1*; adenosine diphosphate, ADP/ATP carrier 1, *LcAAC1*; and sucrose non-fermenting-1-related kinase 2, *LcSnRK2*) were based on the sequences reported in previous study [33]. The real-time quantitative polymerase chain reaction (RT-qPCR) was performed using SYBR^®^ Premix Ex Taq™ II (Tli RNaseH Plus; TAKARA, Dalian, China). The *LcActin* gene (GenBank: DQ990337.1) was employed as a reference gene for normalization. The relative expression levels of the target genes were calculated using the 2^−ΔΔCT^ method.

### 2.5. Quantification of Sugar Content via Gas Chromatography-Mass Spectrometry

The sugar content in litchi pericarp was assayed using gas chromatography-mass spectrometry (7890A-5795C; Agilent, Palo Alto, CA, USA), adhering to the protocol delineated by Wu et al. (2016) [34]. The sugar content was expressed in units of milligrams per kilogram (mg kg^−1^).

### 2.6. Statistical Analysis

The results were presented as mean values with standard error (SE). The *t*-test in SPSS 27.0.1 was used to assess the statistical significance between the experimental group and the control group (* *p* < 0.05, ** *p* < 0.01). Additionally, the Spearman test was utilized for the correlation analysis among BI, MDA content, membrane permeability, respiration rate, ATP content, energy charge, sucrose content, fructose content, glucose content, inositol content, galactose content, and sorbose content, and the correlation heatmap was generated using ggplot2 package within the R programming environment.

## 3. Results

### 3.1. Effects of DNP Treatment on BI, MDA Content, Membrane Permeability, and Respiratory Rate in Litchi Fruit

**Figure 1:**
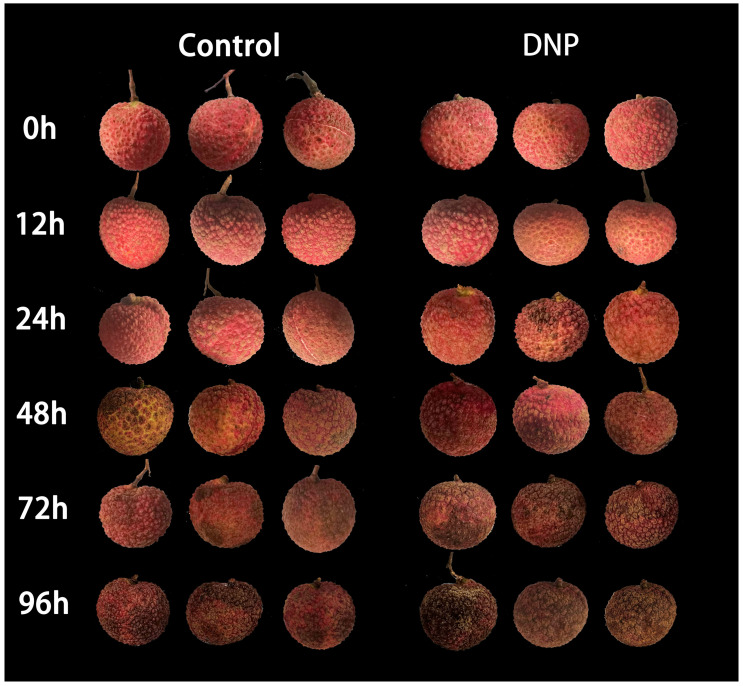
Pericarp browning during storage of litchi fruit in the control and experimental groups.

**Figure 2:**
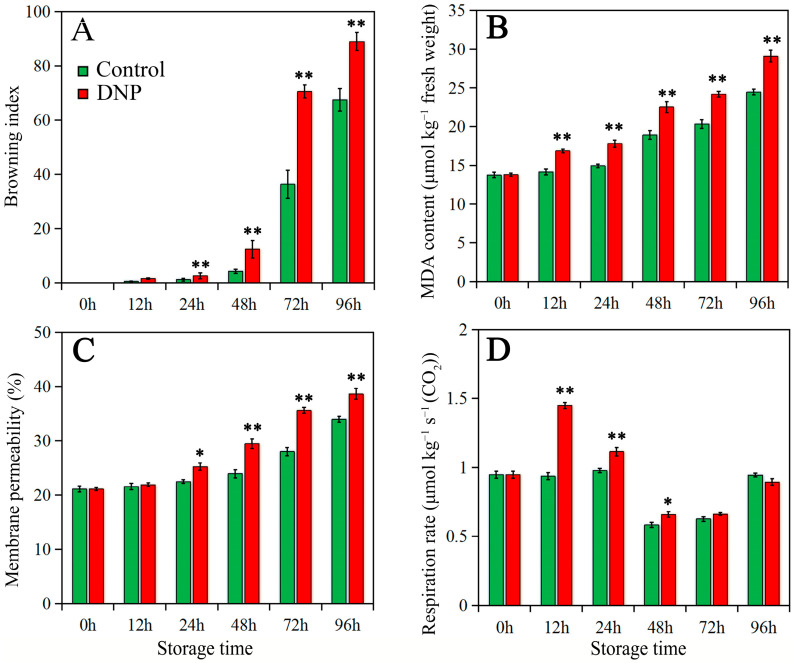
Browning index (**A**), malonaldehyde (MDA) content (**B**), membrane permeability (**C**), and respiration rate (**D**) in fruit of control and DNP treatment during storage. The data was represented as mean ± standard deviation (*n* = 3). The asterisk represents the significant difference (* *p* < 0.05, and ** *p* < 0.01) between the DNP treatment and control groups.

The fruit treated with DNP and the control group showed no obvious browning symptoms before 24 h of storage. Additionally, DNP treatment exacerbated the browning of litchi after 24 h (Figure 1). Figure 2 (panels A, B, C) illustrates that the BI, MDA content, and membrane permeability escalated over time in both the control and DNP-treated fruits during storage. Notably, the DNP-treated fruits exhibited a more pronounced decline in quality, as evidenced by significantly elevated levels of the browning index, MDA content, and membrane permeability compared to the control group following 24 h of storage (*p* < 0.05). After 96 h of storage, the browning index in the DNP-treated fruit surged by 132% relative to the control group. The above results indicated that DNP treatment exacerbated cell membrane damage and deteriorated fruit quality, especially after 24 h.

Furthermore, the respiration rate of the litchi fruits in the control group exhibited a marked decrement during the initial 24–48 h of storage, subsequently followed by a gradual increment (Figure 2D). The DNP treatment amplified the respiration rate of the litchi fruit during the first 72 h of storage, with a particularly pronounced effect in the initial 24-h period.

### 3.2. Effects of DNP Treatment on Energy State in Litchi Fruit

**Figure 3:**
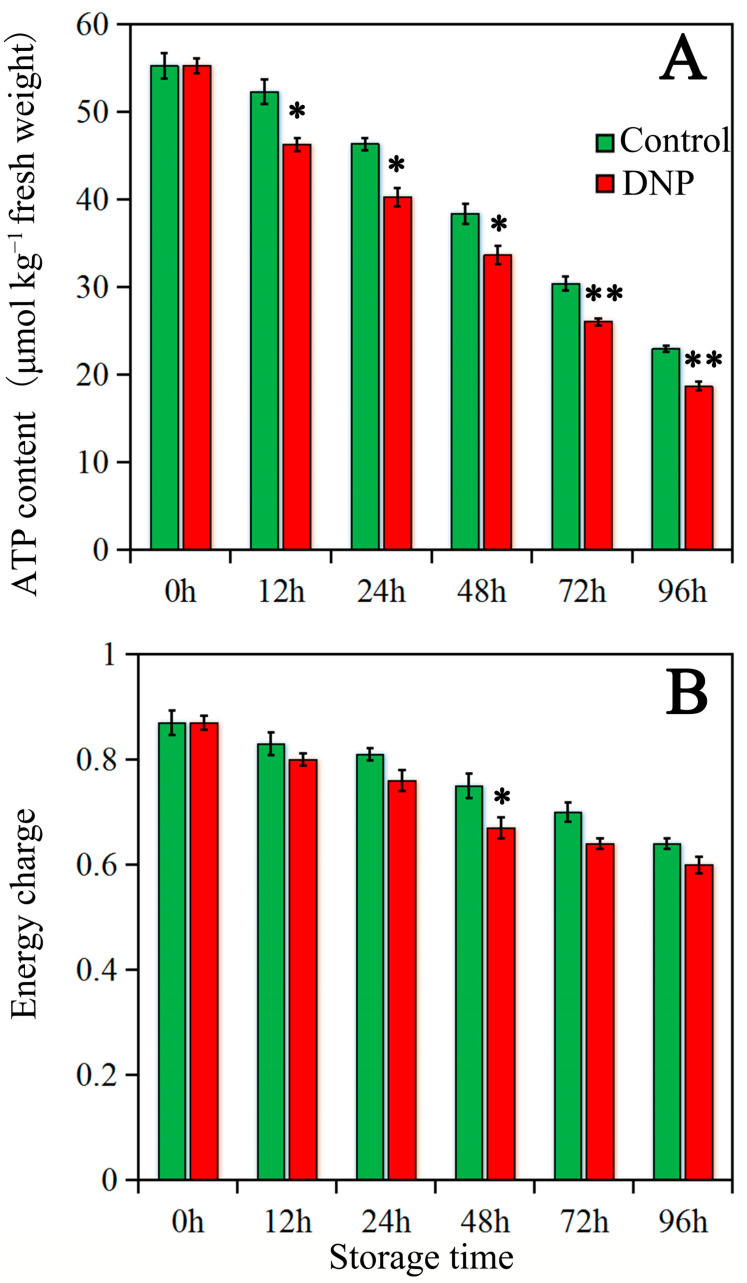
The contents of ATP (**A**) and EC (**B**) in the fruit of control and DNP treatment during storage. The data was represented as mean ± standard deviation (*n* = 3). The asterisk represents the significant difference (* p < 0.05, and ** p < 0.01) between the DNP treatment and control groups.

As the storage duration advanced, the ATP levels and EC in litchi fruit exhibited a gradual decline, as illustrated in Figure 3. The treatment with DNP intensified the decrease in ATP content and EC compared to the control fruit. Particularly, the most substantial decrease in EC was observed at the 48 h during storage, implying that DNP indeed has the effect of reducing the energy state of the fruit.

### 3.3. Effects of DNP Treatment on Expression of Energy Metabolism-Related Genes during Litchi Storage

**Figure 4:**
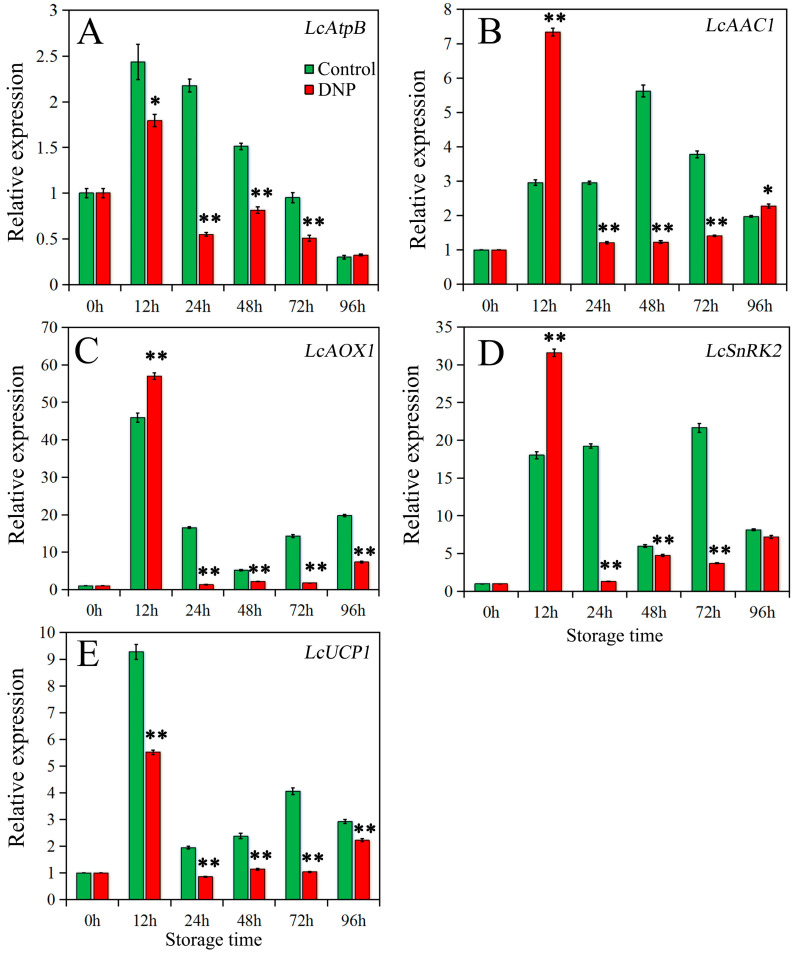
The expression of the energy-related gene ((**A**), *LcAtpB*; (**B**), *LcAAC1*; (**C**), *LcAOX1*; (**D**), *LcSnRK2*; (**E**), *LcUCP1*) in the fruit of control and DNP treatment during storage. The data was represented as mean ± standard deviation (*n* = 3). The asterisk represents the significant difference (* p < 0.05, and ** p < 0.01) between the DNP treatment and control groups.

The relative expression levels of energy metabolism-related genes were depicted in Figure 4. In the control group, the relative expression level of *LcAtpB* rapidly reached its peak within 12 h, which was 2.4 times higher than the initial value (Figure 4A). Subsequently, the expression of *LcAtpB* gradually declined over time. Conversely, the treatment with DNP inhibited the expression of *LcAtpB* in litchi pericarp during storage, resulting in a 26% reduction in peak expression compared to the control. Furthermore, the relative expression level of *LcAtpB* exhibited a rapid decline within 12–24 h, followed by a sustained lower expression level, which differed from the control group. Overall, the expression of *LcAtpB* in litchi fruit was inhibited by DNP treatment during storage.

Regarding *LcAAC1*, in the control fruit, the relative expression level gradually increased and peaked within 48 h, after which it gradually decreased (Figure 4B). DNP treatment markedly promoted early expression of *LcAAC1*, with the peak occurring at 12 h and exhibiting a 30% increase compared to the peak of control. Subsequently, the expression of *LcAAC1* sharply declined within 12–24 h and remained at a low level during the subsequent storage period, indicating that the fruit may have generated an energy demand earlier.

The expression level of *LcAOX1* in both the control and DNP-treated fruit peaked at 12 h of storage (Figure 4C). However, compared to the control, the expression of *LcAOX1* in the DNP-treated fruit was significantly inhibited after 24 h of storage (*p* < 0.01).

The gene *LcSnRK2* in the control fruit exhibited higher expression levels at 12, 24, and 72 h, while lower expression levels were observed at 0, 48, and 96 h (Figure 4D). DNP treatment significantly increased the expression of *LcSnRK2* at 12 h of storage, which was 76% higher than the control. This increase parallels the expression trend of *LcAAC1* observed during the same period. However, DNP treatment inhibited the expression of this gene at 24–96 h.

The expression of *LcUCP1* in the control fruit continued to increase during the early storage period, reaching a level 9.3 times higher than the initial value at 12 h (Figure 4E). However, the expression of *LcUCP1* remained relatively low in the middle to later stages of storage. DNP treatment significantly suppressed the expression of *LcUCP1* during litchi storage, resulting in an average decrease of 30% compared to the control.

### 3.4. Effects of DNP Treatment on Sugar Content Variation in Litchi Fruit

**Figure 5:**
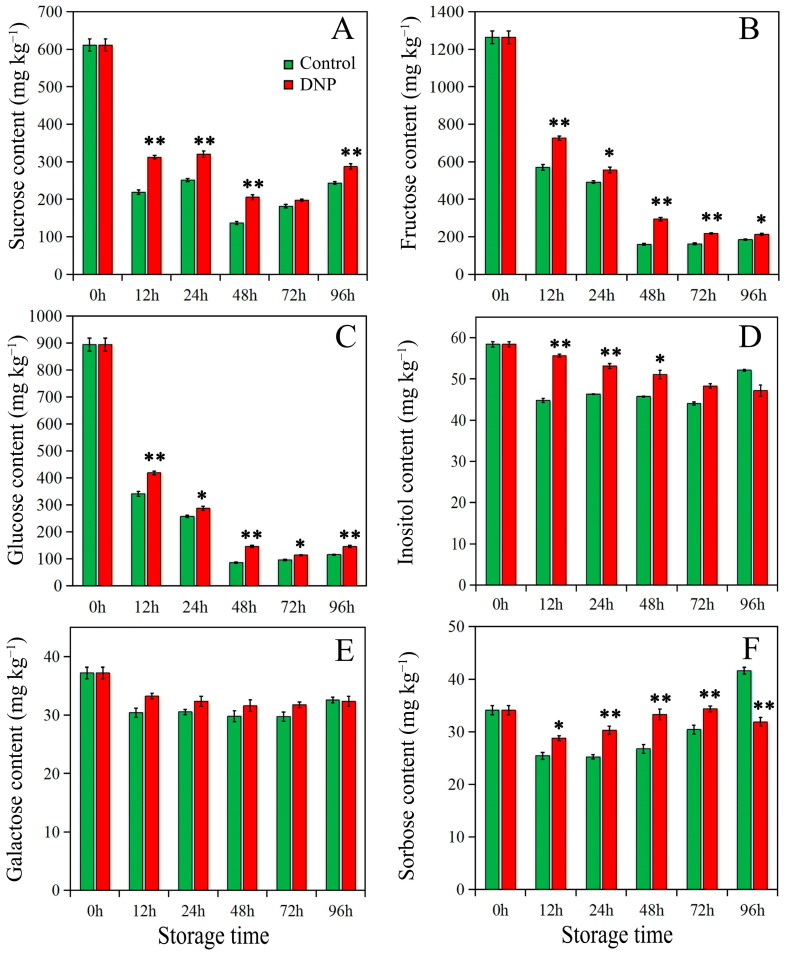
Sugar content ((**A**), sucrose; (**B**), fructose; (**C**), glucose; (**D**), inositol; (**E**), galactose; (**F**), sorbose) in the fruit of control and DNP treatment during storage. The data was represented as mean ± standard deviation (*n* = 3). The asterisk represents the significant difference (* *p* < 0.05, and ** *p* < 0.01) between the DNP treatment and control groups.

In the present investigation, a quantitative analysis was conducted to identify the presence and fluctuations in the concentrations of six carbohydrates in the pericarp of litchi fruit; these carbohydrates include sucrose, fructose, glucose, inositol, galactose, and sorbose (refer to Figure 5). A striking observation was the relative abundance of sucrose, fructose, and glucose in the control group, where their initial concentrations were recorded at 610 mg kg^−1^, 1261 mg kg^−1^, and 895 mg kg^−1^, respectively (Figure 5A–C). Conversely, the concentrations of inositol, galactose, and sorbose were markedly lower, with initial concentrations measuring at 56 mg kg^−1^, 38 mg kg^−1^, and 33 mg kg^−1^, respectively (Figure 5D–F).

Throughout the storage period, a temporal pattern was observed in the concentration levels of sucrose, fructose, and glucose. In the initial 48 h, these concentrations demonstrated a declining trend, which was subsequently followed by a marginal increment between 48 and 96 h. The nadir concentrations recorded were 137 mg kg^−1^, 160 mg kg^−1^, and 86 mg kg^−1^ for sucrose, fructose, and glucose respectively. Intriguingly, when subjected to DNP treatment, the reduction in concentrations of these sugars was impeded during the storage period, with the minimum concentrations manifesting at 72 h.

In contrast, the concentration levels of inositol, galactose, and sorbose remained relatively stable throughout the storage period, with fluctuations being less than 19%, 20%, and 39%, respectively. It is noteworthy to mention that the treatment with DNP led to an elevation in the concentrations of inositol, galactose, and sorbose during the initial 72-h storage phase.

### 3.5. Correlation Analysis

**Figure 6:**
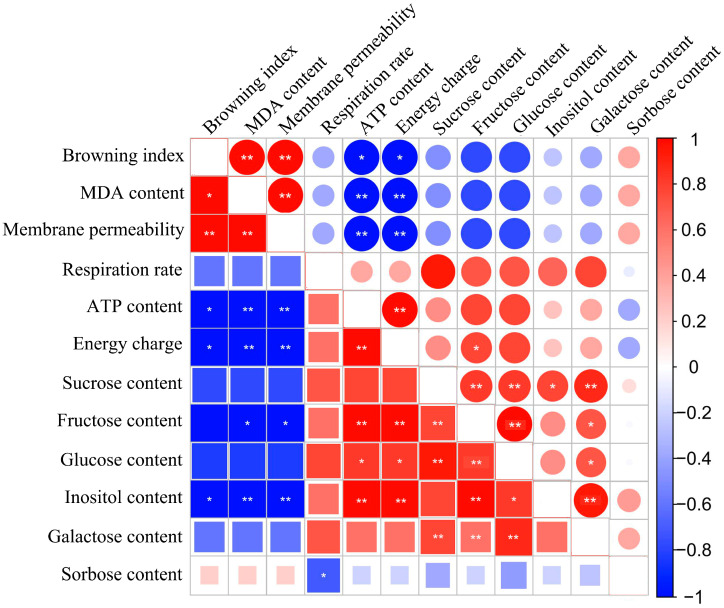
Correlation matrix between the browning index, MDA content, membrane permeability, respiration rate, energy state, and sugar conten. Upper and lower triangles denote the control (circle) and experimental (squar) groups, respectively. Red (+1) indicates the positive correlation and blue (−1) indicates the negative correlation. Asterisk is employed to highlight statistically significant differences (* *p* < 0.05, ** *p* < 0.01).

Correlation analysis, a widely utilized statistical method, can help to reveal the relationships between various physiological and biochemical parameters of postharvest fruit [7]. The correlation analysis between the BI, MDA content, membrane permeability, respiration rate, energy state, and sugar content of litchi was depicted in Figure 6. Our results indicated that the energy charge and ATP content of litchi fruit showed significant negative correlations with the BI, MDA content, and membrane permeability, suggesting that the reduction in energy may exacerbate cell membrane damage. Excluding sorbose, sugars in litchi showed a negative correlation with the BI, MDA content, and membrane permeability, indicating that increasing the content of most sugars may have a protective effect on cell membrane integrity. Notably, inositol in fruit treated with DNP demonstrated a significant negative correlation with BI, MDA content, and membrane permeability (*p* < 0.05).

## 4. Discussion

### 4.1. Effect of DNP Treatment for Energy State Is Related to Postharvest Litchi Quality

As a crop of significant economic value, Litchi chinensis faces a major challenge in the form of rapid pericarp browning, which substantially reduces the fruit’s market value [35]. Postharvest, litchi fruit is invariably subjected to various external stressors, including pericarp desiccation, mechanical damage, and pathogen infestation, which contribute to browning and quality degradation [2,36]. One of the salient features of plant responses to these stressors is the generation of ROS. When produced in excess, ROS can exert deleterious effects on cellular components, including lipid peroxidation, membrane disruption, and the oxidation of proteins and DNA [37,38].

Plants mitigate ROS damage with a dual defense mechanism involving enzymatic and non-enzymatic antioxidant systems. These systems are crucial for regulating ROS levels and lipid peroxidation, and play a key role in increasing antioxidant molecules production [39,40]. Postharvest fruit typically shows increased energy consumption in response to external stressors [41,42]. However, the reduction in energy charge diminishes the effectiveness of the antioxidant systems in litchi fruit, resulting in ROS accumulation within the mitochondria and consequently causing severe damage to cell membrane integrity [43]. Empirical evidence indicates that this damage to cell membrane integrity, along with the subsequent oxidation of phenolic compounds by polyphenol oxidase or peroxidase, is a major cause of postharvest litchi browning [44].

In this study, DNP treatment decreased the energy state within the litchi fruit pericarp, intensifying membrane damage. As a result, there was a significant increase in the browning index and an accelerated senescence in postharvest litchi fruit (Figure 1 and Figure 2A). These findings suggested that the effects of postharvest stressors on litchi pericarp browning are closely associated with the modulation of energy metabolism. The significant negative correlation (*p* < 0.05) between the energy state and pericarp browning further supports this perspective. This study underscores the necessity of developing targeted interventions to mitigate stress-induced energy depletion and associated oxidative damage, thereby extending the postharvest shelf life of litchi.

### 4.2. Expression of Energy Metabolism-Related Genes under Conditions of Energy Deficiency

In litchi fruit, the energy state is closely linked to the expression of energy regulatory proteins, including ATP synthase (ATP synthase β subunit, *LcAtpB*), regulator (*LcSnRK2*), transport (*LcAAC1*), and dissipators (*LcUCP1* and *LcAOX1*) [33]. ATP synthase is an integral enzyme in energy regulation, harnessing the electrochemical ion gradient across membranes to catalyze the synthesis of ATP from ADP and inorganic phosphate (Pi). The β subunit, as a key component of ATP synthase, plays a direct role in ATP synthesis [45]. Recent studies have identified AtpB as a pro-cell-death protein, suggesting that an upsurge in AtpB expression may signify the onset of fruit senescence [46]. Our results indicated that compared to the control group, the expression of *LcAtpB* in litchi treated with DNP was suppressed and peaked at 12 h, and was accompanied by an increase in respiratory rate. This regulation may serve to prevent the ATP synthase from operating in reverse under conditions of diminished proton gradient, thereby avoiding ineffective ATP hydrolysis and promoting ATP synthesis over a short period [47]. Despite the reduction in EC levels due to DNP treatment, litchi fruit showed no significant browning during the first 24 h of storage. This suggests that energy regulation during the initial 24-h period is effective in limiting pericarp browning even under energy-deficient conditions.

SnRK is recognized as an intracellular energy sensor that maintains energy homeostasis by regulating the balance between anabolism and catabolism [48]. Furthermore, SnRK2 has been shown to enhance plant resilience by responding to abiotic stress [49]. Our results indicated that *LcSnRK2* expression significantly increased by 12 h post-DNP treatment, reaching its peak at the same time, which was consistent with the peak expression of *LcAtpB*. This suggests that *LcSnRK2* may work synergistically with *LcAtpB* to boost energy production during energy deficiency [50].

AAC, a mitochondrial membrane transporter protein, facilitates the exchange of cytoplasmic ADP for mitochondrial ATP, thereby catering to the energy requirements of cellular organelles. The transport efficiency of AAC is postulated to be contingent on the mitochondrial membrane potential [51]. The expression of AAC1 in litchi fruit exhibited a rapid escalation within 24 h post-DNP treatment, presumably to accommodate the expeditious generation of energy.

AOX and UCP are widely present energy dissipation systems in plants, play a critical role in energy regulation and stress tolerance in cells [52]. Abiotic stressors may lead to substantial ATP consumption, culminating in an energy crisis [7,53]. Given that ROS generation is an inevitable consequence of mitochondrial aerobic metabolism [54], AOX and UCP serve to sustain the continuous carbon flux requisite for respiratory metabolism, while simultaneously attenuating ROS production, thus stabilizing the tissue’s high-energy state to facilitate various physiological and biochemical reactions [55]. Research indicated that both the AOX and UCP systems were involved in regulating the ripening and senescence processes of postharvest fruit [55,56]. In this study, the expression peaks of *LcAOX1* and *LcUCP1* in DNP-treated fruit were both observed within 24 h of storage, possibly to promote large-scale ATP synthesis and mitigate oxidative damage. In fact, during the first 24 h after DNP treatment, litchi fruit did not exhibit significant pericarp browning, and both MDA content and membrane permeability remained at low levels. Interestingly, *LcUCP1* expression was found to be inhibited at the peak of *LcAOX1* expression, suggesting that *LcUCP1* and *LcAOX1* may not be concurrently engaged in expression regulation at maximal activity during postharvest storage, but rather operate sequentially [57]. After 24 h of DNP treatment, the energy state of litchi continuously decreased, which was accompanied by a significant increase in the browning index and the progressive loss of cell membrane integrity. Meanwhile, the expression of both *LcAOX1* and *LcUCP1* was significantly inhibited. These results suggested that when litchi fruit is unable to maintain the necessary high-energy state, the function of energy dissipation system may be inhibited as an adaptive response to conserve energy and respiratory substrates.

### 4.3. Changes in Sugar Content under Conditions of Energy Deficiency

The responses of plants to external stress are recognized as a high-energy-consuming process [58,59]. During this process, plants utilize various nutrients to generate ATP via metabolic pathways. Sugars are the main substrates for respiratory metabolism, play a significant role in various physiological processes of fruit, such as growth, development, senescence, and stress responses [60,61,62]. Sucrose, fructose, and glucose are the primary forms of sugar storage in litchi fruit [63], which is also confirmed by this study. Our results indicated that DNP treatment resulted in a decreased energy state in litchi, accompanied by a corresponding increase in the content of sucrose and hexoses within the fruit. Correlation analysis revealed a stronger positive link between the levels of sucrose, fructose, and glucose and the energy state in DNP-treated litchi fruits than in the control group, suggesting that the soluble sugar increase might aim to enhance the fruit’s energy state. Research shows that as litchi fruit undergoes senescence and energy loss, its mitochondrial function gradually declines [64]. The pathways of glycolysis and the tricarboxylic acid cycle are gradually impeded, leading to the metabolites in these pathways being progressively diverted to other metabolic pathways, such as the pentose phosphate pathway, fructose and mannose metabolism, amino sugar and nucleotide sugar metabolism, and inositol phosphate metabolism [64]. The changes in the flow direction of metabolites lead to a decrease in the efficiency of carbohydrates in producing energy. To address this issue, litchi promotes the cleavage of polysaccharides (e.g., cellodextrin, starch) in the fruit, thereby increasing the supply of substrates required for respiration. For example, upon sensing an energy-deficient state, SnRK expression can activate the genes of MYBS1 and α-amylase, promoting starch degradation [65]. At the same time, the SnRK expression can enhance plant sensitivity to abscisic acid (ABA) signal [66], which can promote the increase in soluble sugars in cells [67]. These are consistent with the increase in sucrose and hexoses in the fruit following DNP treatment. Therefore, in a low-energy state, litchi might bolster the energy supply by tapping into the fruit’s nutritional reserves to maintain physiological functions, although potentially expediting the decline in fruit quality.

On the other hand, the role of sugars in plant stress resistance has been widely confirmed. For example, fructose and glucose, as key intracellular compounds, can protect cells from damage caused by oxidative stress [68]. Sucrose can maintain the stability of liposomes and has been demonstrated to have stronger antioxidant properties compared to other sugars [69]. However, different species employ distinct strategies of sugar metabolism to combat external stress. For example, blood oranges treated with 24-epibrassinolide maintained higher levels of sucrose, fructose, and glucose, effectively enhanced the fruit’s cold resistance [70]. Apricots treated with oxalic acid exhibited a decrease in sucrose content and an increase in fructose and glucose concentrations, which also helps to inhibit damage to the fruit at low temperatures [71]. This study demonstrated that the levels of sucrose, fructose, and glucose were elevated in litchi fruit treated with DNP, which were inversely correlated with the BI, MDA content, and membrane permeability. This suggested that litchi increased the content of the most abundant sugars under energy deficiency conditions to enhance its antioxidant capacity. It is noteworthy that inositol, a minor sugar in litchi, exhibited a more pronounced negative correlation with pericarp browning, MDA content, and membrane permeability following DNP treatment. Inositol plays a dual role as a signaling molecule and a key metabolite within cells [72]. Research indicates that increasing the content of inositol can enhance the activity of the fruit’s antioxidant system, which helps to counteract the stress effects of biotic and abiotic factors on plants [73,74]. Similarly, some plants also exhibit an increase in sorbose and galactose levels under external stress, such as apple and tomato [75,76]. Therefore, the increase in sugar content in litchi fruit under DNP treatment may be a response to the deterioration of the intracellular environment caused by reduced energy.

## 5. Conclusions

In this study, we elucidated the strategies for litchi to cope with energy deficiency during storage through two experimental conditions—DNP treatment and the control group. The findings suggested that DNP treatment caused energy deficiency in litchi fruit and exacerbated membrane damage and pericarp browning. Under conditions of energy deficiency, litchi fruit can promote energy production by regulating the expression of energy metabolism-associated genes (*LcAtpB*, *LcAOX1*, *LcUCP1*, *LcAAC1*, and *LcSnRK2*), which effectively inhibited pericarp browning within 24 h. Additionally, DNP treatment increased the content of fructose, glucose, inositol, galactose, and sorbose in litchi fruit, presumably as a mechanism to ensure sufficient supply of respiratory substrates and reduce oxidative damage. This suggests that litchi fruit may regulate the senescence process by energy metabolism and sugar metabolism under conditions of energy deficiency. Future research can further elucidate the physiological responses of litchi under energy deficiency by examining the enzyme activity, gene expression and hormonal-signaling in these two key metabolic processes. Our results reveal the mechanisms by which litchi responds to energy deficiency during storage through regulation of energy and sugar metabolisms, contributing to the understanding of the internal regulatory strategies of litchi under stress conditions. These findings provide important reference information for developing innovative postharvest preservation techniques to improve fruit quality.

## Data Availability

The original contributions presented in the study are included in the article, further inquiries can be directed to the corresponding author.
